# M-health: supporting automated diagnosis and electonic health records

**DOI:** 10.1186/2193-1801-2-103

**Published:** 2013-03-12

**Authors:** Efthimios Alepis, Christos Lambrinidis

**Affiliations:** Department of Informatics, University of Piraeus, Piraeus, Greece

**Keywords:** Computer assisted medical diagnosis, Electronic health profile, Medical informatics, M-health

## Abstract

**Background:**

Mobile technology has become a part of our everyday life. Mobile services are used in a wide variety of scientific areas including healthcare. As an intersection of computer supported technology and medicine, m-health is expected to bring higher quality in healthcare. A remedy to deter people from neglecting their health issues is providing further and targeted information, while this information is available on the main devices most people use on a regular basis, namely any station or a mobile phone connected to Internet, enabling access to their health status anytime and at any place.

**Results:**

The authors present a framework that is built based on mobile health and which, in addition, incorporates a module that is responsible for making diagnoses. To achieve this, we have applied the Analytical Hierarchical Process algorithm (AHP) on the test results, making the system able to infer the presence or not of an illness in the subject. Data to be processed emerge from the corresponding subjects’ electronic health records. Through the resulting system, doctors and health companies, which are involved in medical sciences, are offered a sophisticated, powerful tool that provides supplementary diagnoses about their clients by employing their laboratory medical tests.

**Conclusions:**

In this paper a novel computer supported framework is presented, which is targeted basically in the scientific area of mobile health. The incorporated medical diagnosis module and the online presentation of medical tests results may not only facilitate doctors’ and medical agencies work and support healthcare in general, but also and most importantly can benefit users by having an analytical picture of their health status at any place and time. Perhaps one of the most challenging targets for this system to reach is to draw individuals’ attention and give them motives to be more concerned about their health.

## Introduction

In the fast pace of modern life, many of us have caught ourselves at least once in a situation where we were feeling compelled to have a check up, yet something was holding us back and eventually prevented us from doing it. Our everyday activities and responsibilities seem to come forth rather than the concern for our health quality, which seems to be more imperative than our daily routine, if we just consider and the increasing number of health related injuries in urban cities. On top of that, people generally the information they need about health services to make informed decisions about their care.

A remedy to deter people from neglecting their health issues is providing further and targeted information, while this information is available on the main devices most people use on a regular basis, namely any station or a mobile phone connected to Internet, enabling access to their health status anytime and at any place. Ultimately, a time consuming and uneconomical check up becomes a non pestering procedure and also aids people to focus on more salient things, which is their test analyses. Concurrently, an online health test analysis breaks the geographical limitation since doctors and clients are now able to access one’s health status anytime anywhere.

This paper introduces an all web-engineered system that includes three elementary sections. More specifically, it is a web application that communicates with a database where clients’ laboratory medical tests are stored. A medical analysis could be as simple as a check-up or more complexional as an ultrasound. Physicians and medical institutes can upload their clients’ medical tests on the web site, while the registered users can access and view their results when ready and uploaded.

A noteworthy feature of the resulting mobile application is the RSS (Really Simple Syndication)-mobile feature, where medical test results can immediately arrive on user’s mobile display and draw their attention eliminating the need of re-checking for new updates on their healthcare tests’ results.

A sophisticated module of this system is the medical diagnosis that is being conducted all automatically. The automated diagnosis system can diagnose patients with remarkable success assigning a coefficient on each of the risk factors that consist in a medical illness. The algorithm that runs in background is based on the Analytical Hierarchy Process (AHP), an algorithm which is widely used in decision support systems. The Analytic Hierarchy Process (AHP) (Saaty 1980) is composed of several previously existing but unassociated concepts and techniques, such as hierarchical structuring of complexity, pair wise comparisons and an eigenvector method for deriving weights (Liu & Hai 2005).

An automated medical diagnosis system is needful indeed, considering that many physicians come to a medical diagnosis result, based on personal biases. For example, a physician may assess a symptom or a value of a medical test analysis that is more important towards another symptom or a value, while another physician may give a different credit and weight to the specific symptoms and values. This implies that a lot of medical diagnoses cannot be accurate, but also it is a fact that individuals’ medical analyses are susceptible to several interpretations among doctors. This imposes the need of a system that minimizes human bias and considers all relevant and irrelevant data in determining a diagnosis. Also, by having the system online, it is ensured that every doctor has access to the latest scientific diagnoses.

With the Internet gaining more ground among individuals and corporations, it is not uncommon to consider new ways of how the Internet Technology can produce assets for supporting the health care system. Given the fact that an increasingly number of people search medical information over the Web or reconsider the pros and cons of face-to-face medical visits and start to value the “virtual visits”, the Internet has the potential to become a powerful tool as part of a total health care strategy (Oravec 2001). The apparent asset of utilizing the Internet for enhancing the health services offered is the deduction of operating costs for both medical experts and organizations. In addition, the long hours customers spent in waiting rooms can now diminish since the arrival of e-health services. The Internet also provides consumers to check on the qualifications and malpractice claims of the doctors they visit. Yet, there are a lot of steps to be taken to establish and promote the new ways of health care offered to people. Unequivocally, the existing health system has considerable deficiencies, but it would be unrealistic to underpin the idea that on-line health would be without flaws. A proper use of Internet is to use it in conjunction with the advice of certified medical professionals and thus allowing individuals to embrace both kinds of health care.

In view of the above this paper’s resulting system is based on the client–server model and makes use of modern web technology, thus it is accessible from anyone who has Internet access including desktop PC’s and mobile phones. The main extrapolations of this paper are the introduction of a system that auto-diagnoses clients and reports the intelligible medical tests’ results, findings and any diagnosis to them. The system, and particularly the computer aided diagnosis feature, is largely configurable by doctors’ graphical user interface in a way that does not narrow them to the system’s default values, but by allowing alterations on those values so that they reflect each doctor’s expertise and knowledge.

### Related work

In this section we provide works related to the scientific fields of mobile health and computer-aided diagnosis systems. The authors’ work can be seen as the intersection of these two fields.

### Computer-aided diagnosis

Jani & Masri (2011) conducted a research study proposing a system, an online mental status examination that examines individuals’ mental health status in order to determine whether the respective person needs to undergo a more thoroughgoing diagnosis for more specific mental disorders. In detail, individuals make use of web technologies to have their mental health examined. The use of web technologies and also the feedback between clients and doctors while a web application operates as an intermediate among those two parts manifests a strong similarity with our system. Furthermore, the examination results the system extrapolates are used as the primary feedback so that physicians can conduct diagnoses – an essential similarity with our application, which employs medical test results so a diagnosis can be conducted. On the contrary, this research is narrowed to mental health disorders and also there is no consideration about computer-related diagnoses.

Karan et al. (2012) suggested an approach for diagnosing diabetes using neural networks and pervasive computer technologies. The authors value the recent developments in small mobile devices and wireless communications, proposing a system for real time use that operates on a simple client (patient’s PDA) that utilizes neural network for diagnosing illnesses. This system greatly exploits the potential of modern mobile devices while it combines the immediacy, the rapidity and the ease of use on the medical diagnosis.

In a recent publication there is a model proposed that takes advantage of Web-based decision support systems to obtain automatic diagnosis and prognosis for brain tumor. Although this model’s scope is limited to a definite illness, the logic between this model and ours is similar. It refers to an online system that utilizes a web accessible database for storing, managing and sharing biological data related to brain tumors from different clinical centers.

Another mobile health related system is proposed by Bayraktar et al. (2011) that diagnoses internal illnesses. The authors acknowledge the fact that a lot of patients with internal diseases need to undergo many tests at hospitals, which it can be a time consuming and error prone process for patients and doctors respectively. Thus, they developed a system that utilizes a neural-networks algorithm and uses the hospital’s main database in order to diagnose internal illnesses. The produced results, which are simplified and understandable, are then reported to patients. Moreover, the patients may view their analysis results anywhere and at any time using their PDA.

A study was conducted by Wang and Fang (2011) suggesting a remote aided diagnosis system of mental health base. They proposed a system in which the Internet functions as a means, where knowledge base data of mental illness are collected. The produced result is an online database that contains relevant to mental illnesses expert knowledge. This system is world-widely used from anyone having an Internet connection, since doctors have a great amount of mental health related information anywhere at their disposal.

Friedman (2009) conducted a study pointing out the need to utilize computer technology to confront the doctors’ deficiency in rural sub-Saharan Africa. The fact, that there are only few medical institutions in developing countries in general, which is combined with the emigration of large numbers of trained doctors to developed countries and the disease burden, describes a profoundly poor health providence in rural areas. The stride of informatics can offer of hand of assistance and increase the productivity of available doctors. An example of how rural sub-Saharan Africa can find help in information technology is a computer system that was developed in India, the Early Detection and Prevention System (EDPS), and designed for use at rural clinics that do not have a staff physician. It is a decision support system that provides a probable diagnosis and correlating suggestions based on symptoms and signs presented by patients along with other typical information, e.g. height, weight, health history and their source of livelihood. The system, which does not require high-level training or on sight dispensability of nurses or paramedicals, can effectively identify individuals who need immediate attention by health specialists and those who need recurrent treatment.

### Mobile health (m-health)

The reach of mobile technology generated plentiful applications integrated in healthcare. One such application is a mobile controller that gives access to the picture archiving and communication system (PACS) and allows clinicians to manage medical digital images through the mobile phone display (Tang et al.^2004^). There are great opportunities unfolded that can be seized by hospitals and medical agencies, which in turn can facilitate doctors’ work, who now are able to monitor their patients from a distance. The system provides value to existing health care services by remotely and expediently permits access to the patient’s general profile, examination information and associated medical images with no restriction on location as designated hospital facilities are not required.

Mobile computing, medical sensor and communications technologies for health care can all be briefly referred to M-Health. As information systems continue to evolve and advances in wireless communications and mobile hardware further fuel the development and integration of M-Health, challenges arise from mobile health perspective (Istepanian et al. 2004). The quality of health care has been admittedly improved since the advent of the mobile technology, but there are some limitations followed, which mark its future implementation in the provision of health care. The poor linkage of different mobile telecommunication options and standards for e-Health services gives a free ground for more obfuscation on this area. It is also considerable the fact that health-care is a very complex industry and any attempt of re-organizing its mechanisms will be followed by obstacles of non-technological nature. Furthermore, physicians and health workers do not fully understand the latest implemented technologies in health care, since “traditional technology” seems to be enough for certain health services. Mobile applications can positively contribute to the health care field allowing remote e-services with a remarkable reduction in time and cost comparing to existing services for both health workers and individuals. The key question is how existing health institutions can be adjusted in order to fully absorb the benefits of such implementations.

Martinez and Tong (2012) suggested the health e-living concept: Using a mobile display and a sensor, users irrespective of age or geographical location can experience the benefits of healthy living through educative guidelines. Citizens can bolster their wellbeing and improve their health habits by effectively consuming the instructions generated by the mobile application. The system collects data related to exercise activity, food intake and moods that are then delivered to nurse capable of instructing for better health behavior and of keeping user progress on the targeted track. Users realize that personal health is something that is primarily in their control and thereby being proactive about health conditions can be a major contributor to healthcare saving and an alleviator to unnecessary care.

Finally, in (Min 2012), Min introduced an ontology-based personalized disease method employing the machine learning technique and effectively assigning the weight factors to the disease-related variables. The impact of this development is prominent and allows medical checkups and other related services to be delivered without requiring visits to medical agencies. The proposed Personalized Computer Aided Diagnosis Probability (PCADP) algorithm described in (Min 2012) is a personalized statistical disease prediction method. Using the bio signals, types and forms of the physical symptoms and weight factors of a disease that are acquired by the individual as a feedback and applying the embedded components of data management, analysis and decision-making support makes possible to diagnose the presence or not of a disease in the subject.

## Availability and requirements

**Figure 1:**
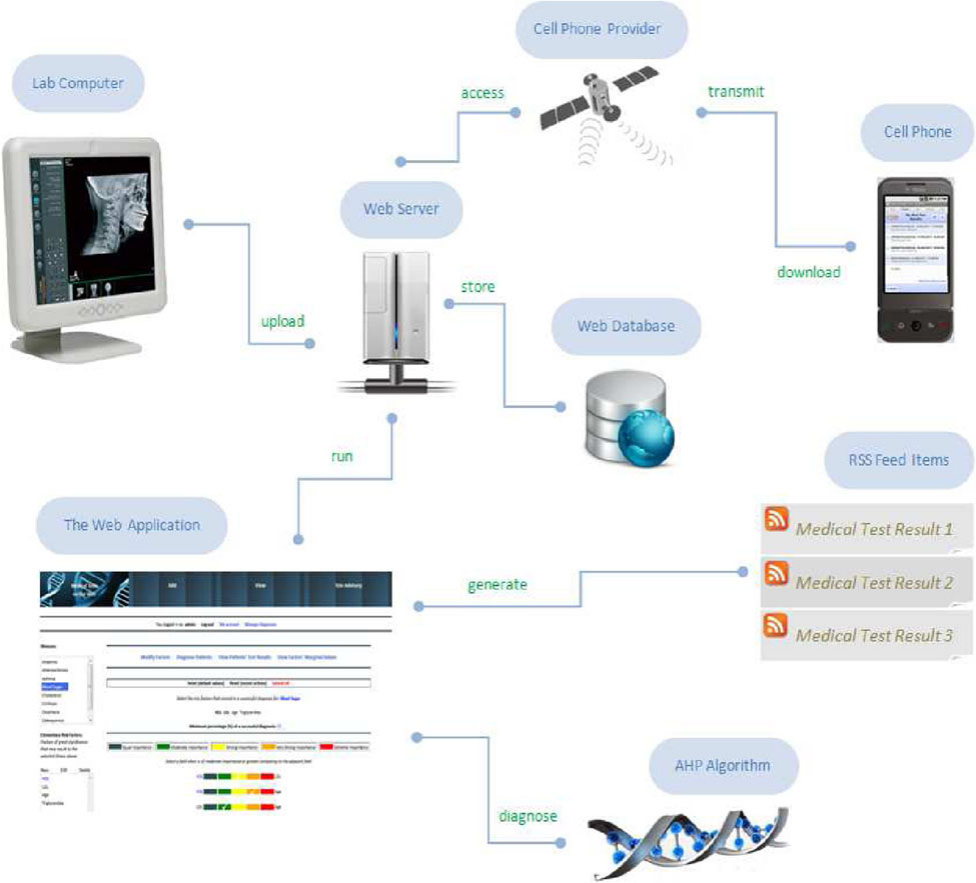
**System’s architecture.**

In this section we give an overview of the system’s general architecture. Figure 1 illustrates an architectural diagram that presents what takes place in the background until the result is presented to the final user. It is shown that Internet acts as the primary means where all the information goes by several levels (items in the figure) until it reaches each user’s cell phone.

A central web server acts as the basic node of the framework’s infrastructure. A database is used to store both all electronic health records as well as data related to the functionality of our algorithmic approach. A web interface is also provided as an alternative and also richer in quality mode of interaction between users and the system.

### Access medical analyses

Accessing one’s health status around the clock may actually motivate them and thus make them become more active about their health issues. This refers to the concept of having an online health status. The procedure of ordering medical tests is much alike the common way except the fact that clients do not have to re-appoint another visit with their reference doctor or the health center to receive the results. The laboratory personnel or the supervised doctor take on uploading clients’ test results on the web site. These test results are stored in a relational database connected to a web server and then are directly retrievable through clients’ GUI (Graphical User Interface).

### RSS mobile-health

Taking the application a level further is the introduction of mobile RSS feature. An RSS feed works by creating a source of data that is machine (computer) readable. RSS uses XML, which stands for eXtensible Markup Language, to encode a variety of information sources in a standardized way. It is very handy for both clients and doctors. The first can be instantly notified and keep track of their lab test results and the latter can be notified about any recently diagnosed patients. Undeniably, the component of mobility could save a lot of time, but also it consolidates the need of automated services conveying all the “stress” from the user to the application. The reason for introducing this feature, besides its arising popularity and the reach of mobile technology in general, is the usability that offers to users by subscribing to several RSS feedbacks and by receiving updates without the web browser requirement-a feature that provides an enhanced linkage between mobile phone and health status.

### The algorithm for automated diagnoses

Besides accessing medical test analyses is the automated diagnosis service using the Analytical Hierarchical Process (AHP) algorithm. One could say that diagnoses that are conducted by the system alone make the physician’s expertise seems obsolete. Whilst, the system offers the physician a form where they can make changes to the contribution extent of the risk factors that consist in an illness or determine the actual risk factors. Therefore, this web application facilitates doctors’ work by providing a quick and effective way of diagnosing patients and giving a lot of assistance to the diagnosis of illnesses so that doctors have a better and more analytical picture of their clients.

As a next step, we describe how the above algorithm works and how the final output is presented to users. Let us consider an example where an individual has their blood sugar tested by a medical organization. The individual’s referenced physician would like to know or be notified whether their patient has hyperglycemia or not. Say the individual’s test results are stored into the system and all that is needed are the contribution extent of each risk factor that consists in hyperglycemia. The risk factors belong to one of the following two categories: the elementary and the secondary factors, in which symptoms are included. The first’s category risk factor values are the actual medical test results, which are used as a feedback for the diagnosis feature. On the other hand, the secondary risk factors can be inferred by a doctor in the context of clinical analysis or can be typical values such as the individual’s age or weight.

Each risk factor is assigned with a number and all risk factors make a sum of 100. The key of AHP is calculating the number or the so called weight each factor is assigned with. That is the pair wise comparisons, the relative importance of one criterion over another that can be expressed. Let us consider for simplicity reasons that only four risk factors consist in a successful diagnosis of hyperglycemia. These are HDL, LDL, Triglyceridea and Age. The first three field values are shown in the latest individual’s test results. The last one is a piece of information which the client provides when registering in the web page. There will be pair wise comparisons between all these risk factors. In our simplified example we have 6 comparisons in total.

The levels of comparisons are five and these are: 1. Equal Importance, 3. Moderate Importance, 5. Strong Importance, 7. Very Strong Importance and 9. Extreme Importance. The majority of the physicians would agree that HDL is of moderate importance compared with LDL, Age is equally important to Triglyceridea and so on. The outcome will be a two-dimension matrix that is populated with the result of the pair wise comparisons (Table 1).

**Table 1:** **Two-dimensional matrix populated with the result of the pair wise comparisons**

	HDL	LDL	Triglyceridea	Age
**HDL**	1/1 (1.00)	3/1 (3.00)	7/1 (7.00)	7/1 (7.00)
**LDL**	1/3 (0.3)	1/1 (1.00)	5/1 (5.00)	5/1 (5.00)
**Triglyceridea**	1/7 (0.14)	1/5 (0.2)	1/1 (1.00)	1/1 (1.00)
**Age**	1/7 (0.14)	1/5 (0.2)	1/1 (1.00)	1/1 (1.00)

The next step is to square the matrix and then compute the eigenvector. The result will be the following (Table 2).

**Table 2:** **The result of squaring the above matrix**

**HDL**	0.582726
**LDL**	0.282482
**Triglyceridea**	0.067395
**Age**	0.067395

This process must be iterated until the eigenvector solution does not change from the previous iteration. This results to a second eigenvector that is virtually identical with the first eigenvector. Iterations prove the validity of the calculations and they are useful when a high precision among the weights is needed. The outcome of the final eigenvector is presented to doctors in a friendlier way, which is each risk factor is now assigned with a percentage, i.e. HDL: 58%.

As a next step, we activate certain rows of the table so the system can conduct a diagnosis. A row is activated when the relevant field in the patient’s test results is beyond a reference range. Say the patient’s test results indicate that HDL and LDL are not within the reference range and are now active because HDL is over 40 mg/dL and LDL is lower than 80 mg/dL considering that the patient is a male. Those two activated rows are then sum up to a positive diagnosis of 80%. Consequently, row activation may take effect if certain fields of the client’s test results are beyond a high or a low reference range. The percentage that consists in a successful diagnosis can be assigned by the physician or the system (default).

As a final step of the algorithm is the immediate notification for the doctor if some of their patients are critically diagnosed.

## Overview of the system

In this section we give an overview of the resulting m-health application. To this end, this section includes a number of screenshots illustrating the resulting system in operation.

**Figure 2:**
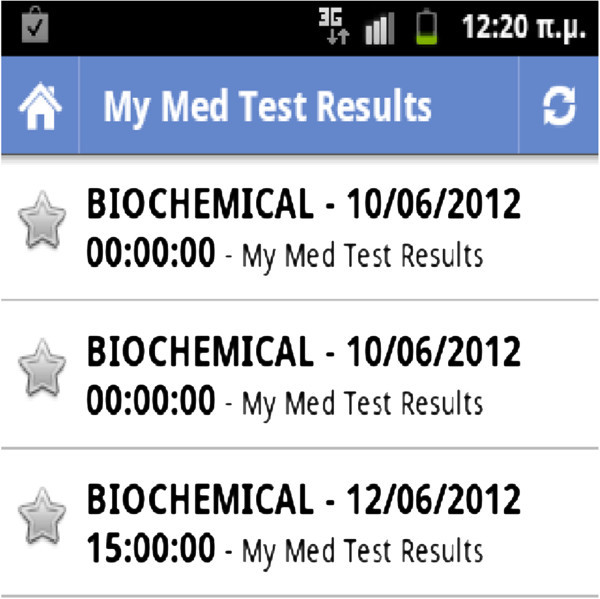
**Viewing the RSS feeds: medical test results.**

Figure 2 illustrates the output of a patient’s medical test results in chronological order. Users may use RSS Reader software of their choice running in the background. Newly uploaded medical test results will pop out the moment they are uploaded. A brief description along with other typical information is shown to the user, which primarily includes various findings and/or reminders that the doctor reckons the client should be aware of.

**Figure 3:**
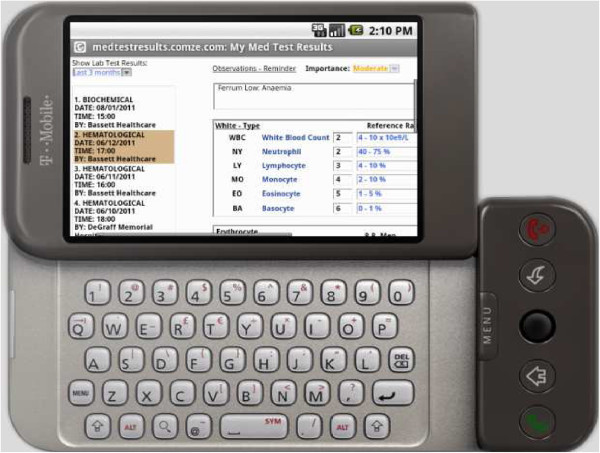
**Medical test results.**

Correspondingly, Figure 3 indicates that the first RSS result was already accessed (clicked down) by the user. The user may use a menu on the left where they can choose a test result among others that they ordered in the past. In each case, the chosen test result appears on the right side.

**Figure 4:**
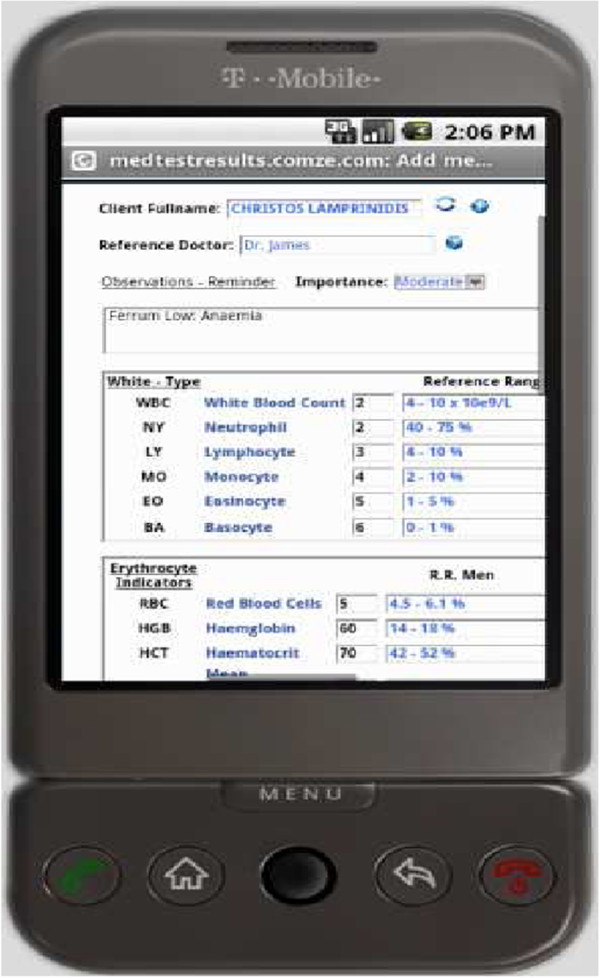
**Preview before uploading a medical test result.**

Figure 4 illustrates a medical test result that is just uploaded to the system, using a mobile device and is waiting for confirmation to be inserted in the database. The file type that was uploaded was an XML file.

**Figure 5:**
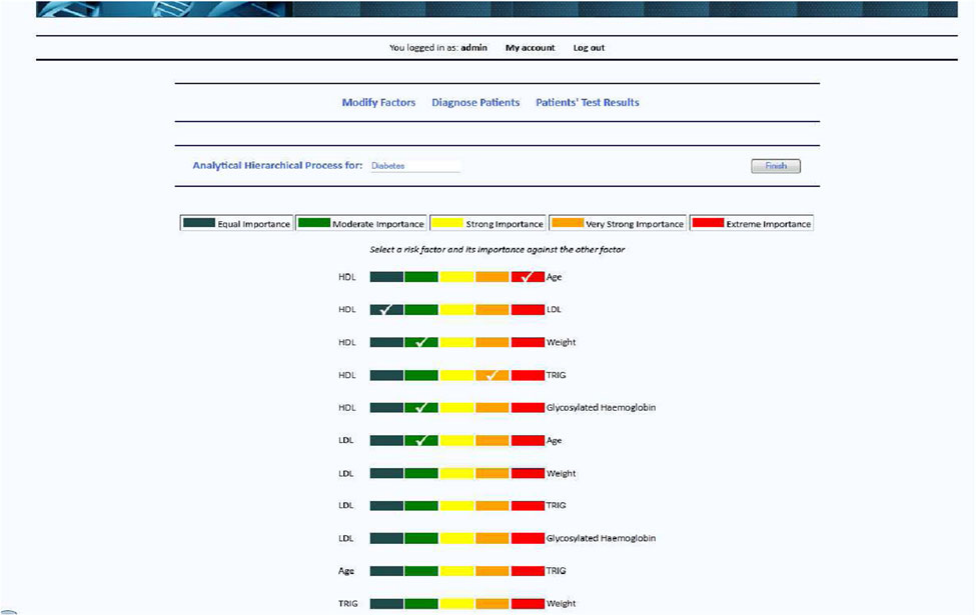
**The automated diagnosis system.**

Figure 5 illustrates an overview of the diagnosis system in operation. Physicians are given a user friendly form where they can make alterations of their choice. On the centre of the page, several actions are provided to the user, such as making pair wise comparisons along with the risk factors.

**Figure 6:**
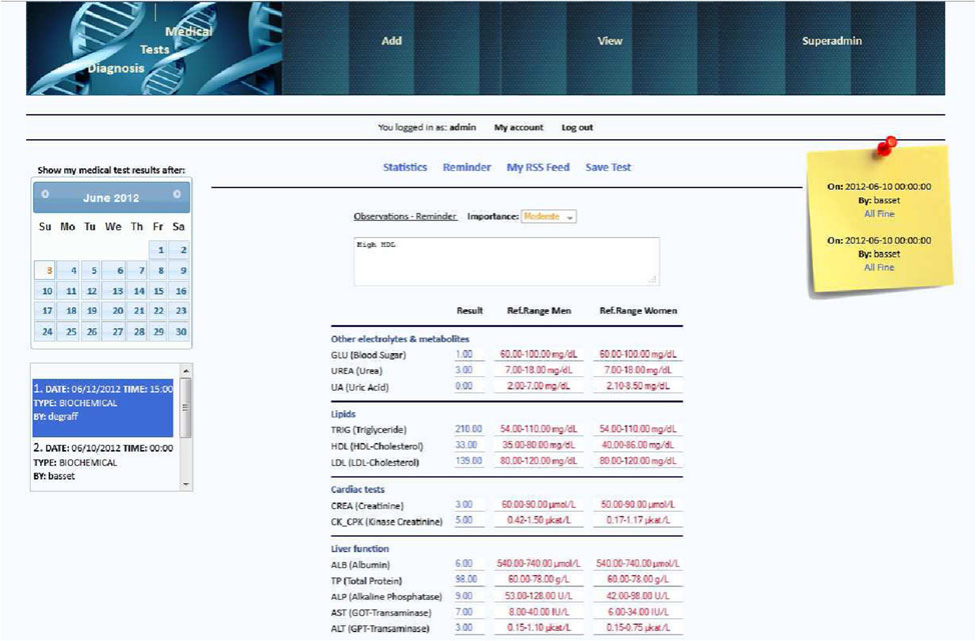
**User medical records and health status messages.**

Finally, Figure 6 illustrates a user’s (as patient) main page, where his/her medical records are accessed. Additionally, as we can see in Figure 6, the system’s diagnosis module is indicating, as a pop up message, that everything considering this specific patient’s health status is fine.

## Conclusion and future work

In this paper a novel computer supported framework is presented, which is targeted basically in the scientific area of mobile health. The incorporated medical diagnosis module and the online presentation of medical tests results may not only facilitate doctors’ and medical agencies work and support healthcare in general, but also and most importantly can benefit users by having an analytical picture of their health status at any place and time. Perhaps one of the most challenging targets for this system to reach is to draw individuals’ attention and give them motives to be more concerned about their health.

A lot of services and spherical aspects could be added to this model so it can even be more beneficial for the clients and make them use an online tool on a regular basis and thereby meet their health care needs. As such, this application supports medical tests that are only text formatted. A more integrated and sophisticated module would be the inclusion of image related health tests. A case in point would be the client’s X-Rays, ultrasounds and MRI’s. Apparently, there are plenty of tests that can be formulated with digital images, which this model does not support, yet these tests are vital in diagnosing. With a software solution, images could be reviewed against known patterns and then presented to a doctor for a final review.

The resulting system requires users to submit the clients’ test analyses to the web site. That suggests that users may have to use the application’s data formats, such as an XML format. Although some supporting formats are popular, especially in the Web field, may not be so renowned for most laboratories’ software. This means that it may be required from users to manually submit tests. This can be a very tedious and long-standing work considering the amount of analyses a health laboratory may have to submit a day. That retracts the concept of receiving one’s test results on the spot. A suggestion would be some kind of integration of the mentioned system and other laboratories’ software.

Tele-advisory would be a helpful feature that would allow doctors and clients having voice/video conferences or text communication in the context of a medical session. This service would be more appealing to people that cannot afford for a regular medical session. It would be also useful for anyone who needs to have a piece of a medical consultation or any other help to an issue that troubles them. The specific feature clearly abolishes any boundaries between doctors and clients and also drastically reduces the cost of appointing sessions with doctors and medical institutes.

Overall, it is hoped this model can be widely applied in the healthcare field since it provides a range of benefits as it combines user friendliness with high utility. At the same time, the greatest barriers would be to gain public and medical institutions support to meet the requirements to fully adopt the electronic medical records and analyses.
